# Applicability of an abbreviated version of the Child-OIDP inventory among primary schoolchildren in Tanzania

**DOI:** 10.1186/1477-7525-5-40

**Published:** 2007-07-13

**Authors:** Matilda Mtaya, Anne N Åstrøm, Georgios Tsakos

**Affiliations:** 1 Centre for international health, UoB, Bergen, Norway; 2 Department of Preventive and Community Dentistry, Muhimbili University College of Health Sciences, Dar es Salaam, Tanzania; 3 Department of Odontology-Community Dentistry, UoB, Bergen, Norway; 4 Department of Epidemiology and Public Health, University College of London Medical School, London, UK

## Abstract

**Background:**

There is a need for studies evaluating oral health related quality of life (OHRQoL) of children in developing countries.

**Aim:**

to assess the psychometric properties, prevalence and perceived causes of the child version of oral impact on daily performance inventory (Child-OIDP) among school children in two socio-demographically different districts of Tanzania. Socio-behavioral and clinical correlates of children's OHRQoL were also investigated.

**Method:**

One thousand six hundred and one children (mean age 13 yr, 60.5% girls) attending 16 (urban and rural) primary schools in Kinondoni and Temeke districts completed a survey instrument in face to face interviews and participated in a full mouth clinical examination. The survey instrument was designed to measure a Kiswahili translated and culturally adapted Child-OIDP frequency score, global oral health indicators and socio-demographic factors.

**Results:**

The Kiswahili version of the Child-OIDP inventory preserved the overall concept of the original English version and revealed good reliability in terms of Cronbach's alpha coefficient of 0.77 (Kinondoni: 0.62, Temeke: 0.76). Weighted Kappa scores from a test-retest were 1.0 and 0.8 in Kinondoni and Temeke, respectively. Validity was supported in that the OIDP scores varied systematically and in the expected direction with self-reported oral health measures and socio-behavioral indicators. Confirmatory factor analyses, CFA, confirmed three dimensions identified initially by Principle Component Analysis within the OIDP item pool. A total of 28.6% of the participants had at least one oral impact. The area specific rates for Kinondoni and Temeke were 18.5% and 45.5%. The most frequently reported impacts were problems eating and cleaning teeth, and the most frequently reported cause of impacts were toothache, ulcer in mouth and position of teeth.

**Conclusion:**

This study showed that the Kiswahili version of the Child-OIDP was applicable for use among schoolchildren in Tanzania.

## Background

Emerging consensus in the literature has identified oral health related quality of life (OHRQoL) as a multidimensional construct containing physical, social and psychological domains [1]. Over the years several socio-dental indicators have been developed, ranging from single item indicators to composite inventories or scoring systems, covering the aforementioned OHRQoL domains [2]. The indices are requested to be simple to use, reliable, valid, precise, acceptable, amenable to statistical analysis, correspond to decision making criteria and to be supported by a relevant theoretical model [3].

Although a number of indices have been developed and tested in population-based studies and studies of patients with specific disorders, most research has been carried out on adults in industrialized countries [4-6]. Yet, there is a lack of OHRQoL measures designed for children and few attempts have been made to evaluate OHRQoL and its determinants in the child populations of non-industrialized countries [7-11]. This is notable, considering that oral disorders are numerous in children globally and likely to affect their quality of life negatively [7]. Untreated dental caries might lead to dental pain which in turn results in impacts of affected play and sleep, avoidance of certain types of food and decreased school performance [12]. Children who have poor oral health have been reported to be 12 times more likely to have restricted activity days than those who do not [12]. A review of studies considering children's self-reported dental pain revealed prevalence rates of 68% in 12-year-old Indians, 42% in 10–14 year-old Ugandans and 21% among 0–18-year-olds in Kenya [13].

Numerous methodological and conceptual problems are involved when developing health related quality of life measures for children; as such measures have to take into consideration distinct changes in the growing child [14]. Most of the changes related to growth may affect the child and therefore the oral health related quality of life measures may have to be age specific. Recently, it has been recognized that using appropriate questionnaire techniques, children can give valid and reliable information and thus should be the primary source of information regarding their OHRQoL [7,15,16]. Two instruments have been developed to measure OHRQoL in younger age groups, namely the Child Perception Questionnaire assessing symptoms, functional limitations and well being in 6–10-year- and 11–14-year-olds and the Child version of the Oral Impacts on Daily Performance (OIDP) inventory [7,8,15,17]. The Child-OIDP, which has been derived from the OIDP [5,18], was developed and tested among Thai school children aged 11–12 yr [7,8]. It has been found to be a reliable and valid instrument when applied to children in Thailand, France and UK [7,8,19,20]. However, further evaluation of its performance across countries and age groups has been requested. As with the adult OIDP, the Child-OIDP measures oral impacts that seriously affect the person's daily life. It is based on the conceptual framework of the World Health Organisation's International Classification of Impairments, Disabilities and Handicaps, ICIDH [21], which has been amended for dentistry by Locker [22]. The OIDP concentrates only on disability and handicap, thus demonstrating strong theoretical coherence and reduced possibility of double scoring of the same oral impacts at different levels [5,18]. Considering respondent burden, the OIDP (and the Child-OIDP) is suitable for use in population surveys, not only in terms of being easier when measuring behaviours rather than feeling states, but also in being short. In order to suit children's cognitive development, the Child-OIDP deviates from the OIDP with respect to the sequence of questions, having a shorter recall period in terms of 3 months instead of 6 months and with pictures used as interviews guide [7]. Since the Tanzanian oral health policy gives priority to children as a target group for oral health care services [23], the Child-OIDP questionnaire is worthy consideration because of its adaptation for use in oral health care needs assessment making it useful for planning services. The original OIDP inventory has previously been translated into Kiswahili and found to be applicable to young adults and older people in Tanzania whilst administered as self-performed questionnaires and in face to face interviews [24,25].

The aim of this study was to assess validity, reliability and prevalence estimates of a Kiswahili translated version of the Child-OIDP frequency inventory for use in primary schoolchildren emanating from two socio-economically different districts in Tanzania.

## Methods

### Study area

A cross sectional survey was conducted in Dar es Salaam, the commercial capital and major sea port of Tanzania, from November 2005 to June 2006. Dar es Salaam is the most densely populated and socially and culturally heterogenic city in Tanzania. According to the 2002 population and house survey in Tanzania, Dar es Salaam has a total population of 2.5 million and population density of 1,793 per square km. Dar es Salaam is divided into three districts; Kinondoni, Ilala and Temeke with total population sizes of 1.083,913, 634,924 and 768,451 people respectively. All districts have drinking water with fluoride content of about 1 mg fluoride/L (1 ppm). Kinondoni and Temeke are quite diverse districts in terms of their socio-demographic profile, with the former having higher employment rates, literacy rates and proportions of the population using the most expensive form, electricity, as their main source of energy for cooking [26].

### Sampling

The study population comprised of children attending standard 7 in public primary schools. A stratified proportionate two-stage cluster sampling design with public primary schools as the primary sampling unit was utilized. To obtain a sample of schoolchildren of mixed socio-economic background, schools were selected at random from urban and rural areas in the Kinondoni and Temeke districts in Dar es Salaam. Overall, 43 rural- (N = 4,809 standard 7 pupils) and 78 urban primary schools (N = 14.725 standard 7 pupils) were listed in Kinondoni. The corresponding number of schools in Temeke were 22 rural (N = 1707 standard 7 pupils) and 77 urban (N = 14103 standard 7 pupils) schools. A sample size of 1200 school children aged 12–14 yr was calculated to be satisfactory for two sided tests, assuming the prevalence of oral impacts to be 0.40 and 0.50 in children with and without caries experience, a significance level of 5%, power of 90% and a design factor of 2 [27]. At the first stage, 4 rural (4/43 n = 755 standard 7 pupils) and 6 urban (6/77, n = 1157 standard 7 pupils) schools in Kinondoni and 1 rural (1/22 n = 184 standard 7 pupils) and 5 urban (5/78, n = 949 standard 7 pupils) schools in Temeke were selected by systematic random sampling using a unified sampling fraction. From a total of 3045 standard 7 pupils available in the selected schools, about 100 students in each selected school (i.e. 1601 students constituting 52.6% of all standard 7 students in the selected schools) and fulfilling the inclusion criteria of being in the defined age range of 12–14 yr were randomly selected from the accessible classes. Only consenting subjects were included in the study and none of the students invited for participation were ill, had a history of psychiatric problems or were disabled. Ethical clearance was obtained from all relevant persons, authorities and committees in Tanzania. These included written permission and clearance for the study from the Research and Publication Committee of the Muhimbili University College of Health Sciences (MUCHS). Permission to work with school children was obtained from Kinondoni and Temeke municipalities, their respective educational authorities, schools administrations, parents and children.

### Translation and adaptation of the Child-OIDP inventory

A structured interview schedule, including the 8 item Child-OIDP inventory was translated from English into Kiswahili, the language of instruction in all Tanzanian public primary schools, by three professionals fluent in Kiswahili and English and back-translated into English by two independent translators. A group of dental professionals reviewed the Kiswahili version of the questionnaire for semantic, experiential and conceptual equivalence with the source version. Sensitivity to culture and selection of appropriate words were considered. The inventory was subsequently discussed and compared with *de novo *oral impacts on daily performances identified in a focused group interview with 10 primary school children. No modifications to scale content and wording were made and the questionnaire was finally pilot tested in a new convenience sample of 63 primary schoolchildren. This confirmed the feasibility of the methodology and helped to determine the time necessary for completion of the interview (about 5–7 minutes). It also led to the decision to avoid pictures as interview guides as well as the severity scales for logistic-, time sparing- and simplicity reasons. In accordance with previous studies that have applied the Child-OIDP inventory [7,8,19], the participants of this study were able to respond to the questions without the aid of pictures and had no difficulty understanding both the content of the questionnaire and any specific words in particular.

### Interview variables

The children completed the Kiswahili version of the Child-OIDP frequency questionnaire at school in face to face interviews administered by two trained research assistants before the clinical examination. The interview started with the children reviewing common oral problems and tick off whether they had experienced them during the previous 3 months [7,8]. The Child-OIDP frequency index referred to difficulty carrying out eight daily life activities namely eating, speaking, cleaning mouth, sleeping, smiling, school work, emotion and social contact each scored 0–3 where (0) never, (1) once or twice a month, (2) once or twice a week, (3) very day/nearly every day [7,8]. Participants were also asked to identify the oral condition that caused the specific impacts by answering for each reported item (1) yes or (0) no to the following alternatives: "toothache, sensitive teeth, tooth exfoliation, problems with position of teeth, ulcer in mouth, bleeding in mouth, swollen gums, bad breath, problems with colour of teeth, problems with spaces of teeth, other problems". The total Child-OIDP score was constructed in two ways. First, by adding the 8 performance scores as originally scored (0–3) into a Child-OIDP additive score (ADD) (range 0–24). Second, the Child-OIDP simple count (SC) score (range 0–8) was constructed by summing the dichotomized frequency items of (1) affected and (0) not affected.

The predictor variables and the number of subjects according to categories are summarized in Table 1. Socio-demographics were assessed in terms of place of residence (urban/rural), district (Kinondoni/Temeke), gender, age and parental education. A group variable on parental education was constructed from two dummy variables (0/1) on father's and mother's highest level of education. Self reported oral health status, satisfaction with teeth/mouth and self rated health status were coded on 4-point Likert scales and recoded further into dummy variables in terms of (0) good/satisfied and (1) bad/dissatisfied. Overall satisfaction with teeth was constructed as a sum variable from 4 variables (satisfaction with mouth/teeth, position of teeth, appearance and colour of teeth) and dichotomized for use in cross tabulation and logistic regression analysis. Frequency sugar intake was made up by a sum score of items assessing the frequency intake of biscuits, chocolate/toffee/sweets, ice cream, soda, and sugared fruit juice. Each item originally assessed on a scale ranging from (1) more than once a day to (4) seldom or never was dichotomized into (1) (categories 1,2) and (0) (categories 3,4); then, the scores of those derived variables were again summed and dichotomized. Dental attendance was constructed into two Yes (1) and No (0) variables originally scored from (1) attended more than 3 times to (5) never attended. Sucking behaviour, including finger, lip tongue sucking was scored as Yes (1) and No (2) variables.

**Table 1:** Frequency distribution of independent variables and their categories according to district

Variables	Categories	Kinondoni % (n)	Temeke %(n)	p-value
Sex	Male	41.1 (412)	36.8 (220)	P = 0.050
	Female	58.9 (591)	63.2 (378)	
Age	12 Yrs	26.1 (262)	23.9 (143)	P = 0.033
	13 yrs	41.9 (420)	48.5 (290)	
	14 yrs	32.0 (321)	27.6 (165)	
Parental education	Both low	38.5 (210)	53.8 (149)	P = 0.000
	One low/one high	24.2 (132)	20.9 (58)	
	Both high	37.2 (203)	25.3 (70)	
Place of residence:	Urban	63.5 (637)	82.3 (492)	P = 0.000
	Rural	36.5 (366)	17.7 (106)	
DMFT	0	78.3 (785)	77.6 (464)	P = 0.399
	> 1	21.7 (218)	22.4 (134)	
OHIS Debris score	Good	68.0 (682)	61.9 (370)	P = 0.007
	Fair/poor	32.0 (321)	38.1 (228)	
Overall satisfaction	Satisfied	87.8 (881)	90.1 (539)	P = 0.092
with oral health	Dissatisfied	12.2 (122)	9.9 (59)	
State of teeth	Good	84.8 (851)	91.6 (548)	P = 0.000
	Bad	15.2 (152)	8.4 (50)	
State of health	Good	93.2 (935)	96.5 (577)	P = 0.003
	Bad	6.8 (68)	3.5 (21)	
Oral problems	None	43.3 (434)	30.1 (180)	P = 0.000
	≥1	56.7 (569)	69.9 (418)	
Finger sucking	No	70.6 (708)	66.1 (395)	P = 0.033
	Yes	29.4 (295)	33.9 (203)	
Dental attendance	No	86.8 (871)	80.9 (484)	P = 0.001
	Yes	13.2 (132)	19.1 (114)	
Sugar intake	0–1 item	35.3 (354)	36.5 (218)	P = 0.339
	> 1 items	64.7 (649)	63.5 (380)	

### Clinical Examination

One trained and calibrated dentist (MM) conducted all clinical examinations in the classroom setting with natural daylight as the source of illumination and with an assistant recording the observations. Participants identified with problems that needed treatment were referred or advised to seek treatment at the two municipal hospital of Kinondoni and Temeke districts and oral health education sessions were provided. Caries experience was assessed in accordance with the WHO criteria [28]. Oral hygiene was assessed using the simplified-Oral Hygiene Index (OHI-S) [29]. Duplicate clinical examinations were carried out on a randomly selected sub-sample of 71 participants considered to be representative of the study subjects. Analyses performed on the duplicate examination recordings gave kappa statistics of 0.93 and 0.74 for the DMFT- and OHI-S scores, respectively. These figures indicate very good intra-examiner reliability [28].

### Statistical analyses

Test-retest reliability for the clinical parameters and the questionnaire variables was assessed using Cohen's weighted kappa statistics with an independent convenience sample of 60 12–14-year-olds and a time interval of 1.5 weeks. Internal consistency reliability was assessed in the main sample using Cronbach's alpha [30]. Construct validity was determined by comparing OIDP scores of groups that differ regarding subjective measures of health status. Furthermore, differences in Child-OIDP were also assessed between groups according to socio-economic-, clinical and behavioral characteristics. Construct validity was also evaluated using exploratory factor analysis, EFA (i.e. Principle Component Analysis with Varimax rotation) with the independent sample constituting the test-retest group- and confirmative factor analysis, CFA with the main sample. The parameters of CFA were estimated with maximum likelihood estimation (ML) and bootstrapping advocated for non-normally distributed variables [31]. Bias corrected 90% CI (SE/BC 90% CI) was reported for the estimates. Adequacy of the model fit was assessed using chi-square statistics, the Goodness of Fit index (GFI), the Incremental Fit Index (IFI), the Normed Fit Index (NFI) and the Comparative Fit Index [31]. Cross-tabulation and chi-square statistics were used to assess bivariate relationships. Multivariate analysis was done by Logistic regression. For the purpose of cross tabulation and logistic regression analysis the OIDPSC score (0–8) was dichotomized as 0/1+, producing the categories (0) "no daily performance affected" and (1) "at least one daily performance affected". The distribution of the OIDPSC scores supported this cut-off point. Data were analyzed using SPSS version 14.0 and AMOS 6.0. To adjust for the effect of the cluster design, data were reanalysed using STATA 9.0 with survey command. P-value for statistical significance was set at 0.05.

## Results

### Sample profile

A total of 1003 children from Kinondoni (63.5% urban, 58.9% girls, mean age 13.1 yr) and 598 children from Temeke (82.3% urban, 63.2% girls, mean age 13.0 yr) completed an extensive personal interview and underwent a full mouth clinical examination. The mean DMFT scores were 0.37 (sd = 0.86) and 0.39 (sd = 0.84) in Kinondoni and Temeke, respectively. Corresponding scores concerning OHI-S were 1.0 (sd = 0.53, range 0.0–3.3) and 1.2 (sd = 0.54, range 0.0–4.2). Table 1 provides the percentage distribution of participants' socio-demographic-, clinical-, perceived oral health- and behavioral characteristics in the total sample and according to district of residence.

### Reliability and validity of the Child-OIDP

All the participating subjects completed the Child-OIDP frequency inventory providing support to its face validity. Internal consistency reliability (standardized item alpha) was .77 (.62 in Kinondoni, .76 in Temeke). The inter item correlations ranged from 0.05 (speaking/carrying out major work) to 0.79 (speaking/contact with people). The corrected item total correlation (i.e. the correlation between each item and the total score omitted for that item) ranged from .21 (carrying out major work) to .69 (contact with people) being above the minimum level of 0.20 for including an item into a scale [30]. The Cronbach's alpha decreased when any one item was deleted from the scale except for the items of emotion and schoolwork. Test-retest reliability of the 8 categorical Child-OIDP items in terms of weighted Cohen's kappa were 0.7 (emotional state), 0.8 (carrying out major schoolwork) whereas eating, speaking, cleaning teeth, sleeping, smiling and social contact showed a kappa value of 1.00. Weighted Cohen's kappa for the categorical Child-OIDPSC scores were 0.91 (1.0 in Kinondoni and 0.83 in Temeke) and intraclass correlation coefficient for Child-OIDPADD scores were 0.98.

Construct validity was demonstrated in that the Child-OIDP scores increased as the children's self-reported oral health-, general health-, dental appearance- and oral problems status changed from healthy to unhealthy. This was evident with Chi-square test in cross-tabulation analyses and with Mann Whitney U test using the Child-OIDP SC and the Child-OIDP ADD scores, respectively (Table 2). Children that were not satisfied with their oral health and rated their teeth status as bad had mean Child-OIDP ADD score at least twice as high as that of children who felt satisfied and rated their teeth status to be good (Table 2). The Child-OIDP SC and the Child-OIDP ADD scores also varied with socio-demographic variables (Table 3).

**Table 2:** The Child-OIDP scores and self-reported and clinically assessed variables. Percent of children with Child-OIDP > 0 and mean Child-OIDP scores with differences in mean rank, DMR, (Mann Whitney U test).

Self rated oral health	% Child OIDPSC > 0	Mean (SD) Child OIDP ADD scores	DMR
*Overall satisfaction with teeth*			
Satisfied	26.0 (369)	1.0 (2.5)	
Dissatisfied	49.2 (89)**	2.4 (3.8)**	91.1
*State of teeth*			
Good	25.1 (351)	1.0 (2.6)	
Bad	53 (107)**	2.3 (3.4)**	120.5
*State of general health*			
Good	27.6 (417)	1.1 (2.7)	
Bad	46.1 (41)**	1.9 (3.3)**	38.0
*Reported oral problems*			
No problem	11.6 (71)	0.4 (1.7)	
≥1 problems	39.2 (387)**	1.6 (3.1)**	256.2
*DMFT*			
DMFT = 0	26.7 (333)	1.1 (2.6)	
DMFT > 0	35.5 (125)*	1.5 (3.1)**	59.5
*Oral Hygiene score*			
Good	27.3 (287)	1.1 (2.7)	
Fair/poor	31.1 (171)ns	1.2 (2.8) ns	34.1

**p < 0.001, *p < 0.05

**Table 3:** The Child OIDP and socio-demographic and behavioral variables. Percent of children with OIDP > 0 and mean OIDP scores with differences in mean rank (Mann Whitney U test).

Socio-behavioral variables	% OIDP > 0	Mean (SD) OIDP ADD scores	DMR
*Place of residence*			
Urban	28.3 (319)	1.1 (2.7)	
Rural	29.4 (139)ns	1.2 (2.7) ns	9.7
*Age*			
12 yrs	24.0 (97)	1.1 (2.8)	
13 yrs	30.8 (219)	1.2 (2.5)	
14 yrs	29.2 (142)*	1.3 (2.9) ns	37.6
*District*			
Kinondoni	18.5 (186)	0.4 (1.2)	
Temeke	45.5 (272)**	2.3 (3.9)**	247.1
*Finger sucking*			
No	25.8 (285)	1.0 (2.7)	
Yes	34.7 (173)**	1.4 (2.9)**	74.8
*Dental attendance*			
No	26.5 (359)	1.1 (2.6)	70.1
Yes	40.2 (99)**	1.8 (3.4)**	
*Sugar intake*			
0–1 item	25.2 (144)	1.1 (2.9)	
> 1 items	30.5 (314)*	1.2 (2.6)*	48.1

** p < 0.001. *p < 0.05

By examining the relationship between Child-OIDP SC and clinical and non-clinical variables in a single regression model, possible confounding due to strong associations between the explanatory variables was taken into consideration (Table 4). Socio-demographic and behavioral variables were entered in the first step with model summary in terms of Nagelkerkes R^2 ^= .147 and with all variables being statistically significantly associated with Child-OIDP. By entering the DMFT and OHI-S status in step 2, the model summary increased to Nagelkerkes R^2 ^= .152. Variables on self-reported oral health entered in step 3 raised the model summary to Nagelkerkes R^2 ^= .301. In the final model (Model Chi Square = 377.006, df = 14, p = 0.001), district of residence (Kinondoni/Temeke), area of residence (urban/rural) and reported satisfaction with oral health, status of teeth, status of health and reported number of oral problems remained highly statistically significant predictors with odds ratios of 4.8, 1.6, 1.9, 3.1, 1.8 and 3.9, respectively. Interactions between district and DMFT status (B = -0.704, p = 0.016) and between districts and sugar frequency intake (B = -0.780, p = 0.005) upon Child-OIDP scores were revealed indicating that the tendency of children with caries experience and frequent sugar intake to be more likely than their counterparts without those characteristics to report any oral impact (Child-OIDP > 0) was evident in Kinondoni but not in Temeke district.

**Table 4:** Unadjusted and adjusted odds ratios and 95% confidence interval (CI) of having at least one oral impact on daily performances (OIDP = 1) according to non-clinical and clinical variables.

	Unadjusted OR (95% CI)	Adjusted OR (95% CI)	p-value
Step 1			

*District*: Kinondoni	1	1	P = 0.001
Temeke	3.6 (2.9–4.5)	4.8 (3.6–6.2)	
*Area*: Urban	1	1	P = 0.001
Rural	1.0 (0.8–1.3)	1.6 (1.2–2.2)	
*Sex*: Male	1	1	P = 0.736
Female	0.9 (0.7–1.1)	0.9 (0.7–1.2)	
*Age*: 12 yrs	1	1	P = 0.052
13 yrs	1.4 (1.1–1.8)	1.3 (1.0–1.7)	
14 yrs	1.3 (0.9–1.7)	1.2 (0.9–1.7)	
*Finger/lip sucking*: no	1	1	P = 0.093
yes	1.5 (1.2–1.9)	1.2 (0.9–1.6)	
*Sugar items*: 0–1	1	1	P = 0.207
> 1	1.3 (1.0–1.6)	1.2 (0.9–1.5)	
*Dental attendance*: no	1	1	P = 0.062
yes	1.8 (1.4–2.7)	1.3 (0.9–1.9)	
Step 2			
DMFT = 0	1	1	P = 0.238
DMFT > 0	1.5 (1.2–1.9)	1.2 (0.8–1.6)	
Step 3			
*OHIS*: good	1	1	P = 0.916
fair/poor	1.2 (1.0–1.5)	1.0 (0.7–1.3)	
Satisfied with oral health	1	1	P = 0.001
Dissatisfied with oral health	2.8 (2.0–3.7)	1.9 (1.3–2.8)	
No oral problem	1	1	P = 0.001
≥1 oral problems	4.9 (3.7–6.5)	3.9 (2.9–5.2)	
*State of teeth*: good	1	1	P = 0.001
bad	3.3 (2.4–4.5)	3.1 (2.1–4.5)	
*State of health*: good	1	1	P = 0.023
bad	2.2 (1.4–3.4)	1.8 (1.1–3.0)	

EFA gave 3 factors with eigenvalue greater than 1. The factors accounted for 71.7% of the Child-OIDP variables. Factor 1 consisted of "speaking" "smiling" and "social contact" (loading higher than 0.4), Factor 2 consisted of "eating" and "cleaning" (loading above 0.4) and Factor 3 consisted of "emotion" and "schoolwork", all with factor loadings above 0.4. "Sleeping" loaded highly (loading 0.5) on both Factor 2 and 3. CFA was then used to test the hypothesized 3-factor (social function, physical function, psychological function) model identified from the EFA which is consistent with previous experience [32]. The four indices from CFA indicated acceptable fit of the model with the data in terms of GFI (0.96), AGFI (0.93). NFI (0.95) and CFI (0.96). However, the Chi -square was statistically significant (Chi-square = 221.137 (17), p < 0.001) and the RMSEA (0.08) a bit below the optimal level of 0.05 indicating a mediocre fit. The amount of variance (R^2^) accounted for was 26% (.012/0.24–0.28), 32% (.03/0.28–0.37) and 4% (0.04/0.03–0.05) by the social (speaking, smiling, contact with people), functional/physical (eating, cleaning) and psychological (emotion, schoolwork, sleeping) dimensions, respectively. The present model appeared to provide the "best" description of the Child-OIDP data in this study when compared with a 2-factor and a 1-factor model.

### Child-OIDP prevalence and the perceived causes of oral impacts

**Figure 1:**
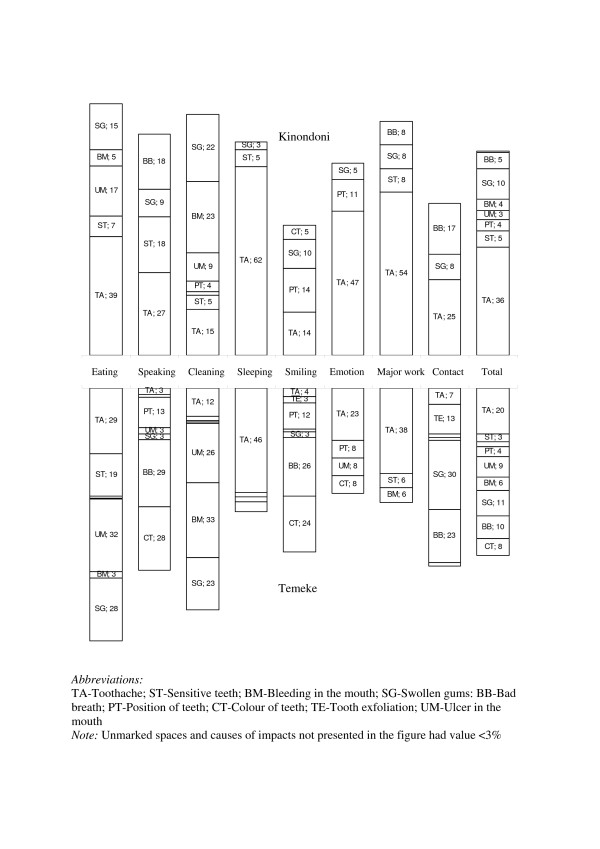
Perceived oral problems associated with oral impacts in schoolchildren from Kinondoni and Temeke districts.

The most common oral problems in both districts initially listed by the schoolchildren were toothache (Kinondoni: 18.7%, Temeke: 24.1%), having ulcer in the mouth (Kinondoni: 15.7%, Temeke: 26.6%), bleeding (Kinondoni: 15.4%, Temeke: 17.4%) and swollen gums (Kinondoni: 18.1%, Temeke 21.4%). The mean Child-OIDP ADD scores for the total sample showed limited variability with a mean of 1.2 (sd= 2.8). Overall, a total of 28.6% (95% CI: 26.6, 30.6) had at least one oral impact. As can be seen from Table 5, the prevalence of oral impacts was moderate in Kinondoni (18.5%; 95% CI: 16.5, 20.5) but relatively high in Temeke (45.5%; 95% CI: 42.5, 48.5). Impacts on eating were the most prevalent reported impairment in Temeke (35.3%), followed by cleaning teeth (26.9%) and smiling without embarrassment (13.0%). The corresponding figures in Kinondoni were eating (13.3%) and cleaning teeth (8.6%), with all other activities showing very low levels of oral impacts. Impacts on emotion and school work were the least frequently reported impacts in both districts. As shown in Figure 1, toothache was the most frequently perceived cause of impairments for almost all performances particularly in Kinondoni. In Temeke, the majority of impacts on speaking and smiling were attributed to bad breath, colour of teeth and position of teeth. Swollen gums, bad breath and bleeding in mouth were frequently perceived causes of impairments in both districts.

**Table 5:** Percentage distribution of the eight Oral Impacts on Daily Performance (OIDP) frequency items in the whole sample (n = 1601) and by district of Kinondoni and Temeke.

Child – OIDP performance items	All % (n)	Kinondoni % (n)	Temeke % (n)
Eating	21.5 (344)	13.3 (133)	35.3 (211)
Speaking	5.4 (86)	1.1 (11)	12.5 (75)
Cleaning teeth	15.4 (247)	8.6 (86)	26.9 (161)
Sleeping/relaxing	5.3 (85)	3.7 (37)	8.0 (48)
Smiling	6.2 (99)	2.1 (21)	13.0 (78)
Emotional	2.0 (32)	1.9 (19)	2.2 (13)
School work	1.8 (29)	1.3 (13)	2.7 (16)
Enjoying contact with people	5.1 (81)	1.2 (12)	11.5 (69)
% with at least one OIDP	28.6 (458)	18.5 (186)	45.5 (272)

## Discussion

The present study is the first large population based survey about OHRQoL covering schoolchildren in Tanzania. Comparison of the sample characteristics with the Kinondoni and Temeke child populations on the markers of sex and parental education suggest that the sample was representative of the population of children aged 12–14 yr in those districts. When administered in face to face interviews, the Kiswahili version of the inventory showed good reliability and validity in 12–14-year-old schoolchildren, thus indicating its applicability. The internal consistency reliability was successfully tested in several ways. All inter-item correlations were positive and all corrected item total correlations were above the minimum level of 0.20 for an item to be included into a scale [30,33]. Cronbach'a alpha was 0.77 which is satisfactory according to the standards of 0.50 and 0.70 thresholds set by most authors for group comparisons [33]. These figures compare in magnitude with those reported for the Thai-, UK- and French version of the Child-OIDP inventory [7,8,19,20]. Previous applications of OIDP among adolescents and young adults in various populations in Africa have yielded higher internal consistency values ranging from 0.70 to 0.91 [34,35].

All participating children completed the 8 item Child-OIDP inventory adding support to the face validity of its Kiswahili version. There was no indication from the reference groups of academics or from the focus group discussions and pilot surveys that the relevance of any of the items was low in the Tanzanian context. This suggests that Tanzanian schoolchildren were capable of fully understanding the Kiswahili translated version without altering the meaning of the questions and that the Kiswahili and English frequency inventories are comparable. Whereas face and content validity was assessed by a ground up or *de novo *approach based on qualitative focus group interviews with the targeted children, hypotheses regarding the construct validity were confirmed in that the inventory varied systematically and in the expected direction with self-reported oral health indicators. Thus, the Child-OIDP scores indicated lower levels of oral impacts when the self-perceived oral health was better and when no oral problems were recorded.

By examining the relationships between the Child-OIDP scores and clinical-, non clinical and socio-behavioral variables in a single regression model, it was possible to obtain a better understanding of their combined effects and to compare the strength of the influence from each. Consistent with previous findings and with the propositions derived from the ICIDH conceptual framework [21], the present results suggest that adjacent concepts such as oral health perceptions and reported oral problems were the strongest predictors of oral impacts. In contrast, the effects of the more remote concepts of clinical scores of DMFT- and OHI-S did not remain statistically significant in the multivariate analyses, suggesting that their effects were mediated through other variables. Previous studies conducted in Tanzania have shown that the OIDP frequency scale is able to discriminate between subjects with and without at least one clinically defined problem, and between older adults with complete and reduced number of posterior occluding support [24,25]. Bivariate associations between the Child-OIDP scores and number of decayed primary and permanent teeth were recently reported among French children [19].

The more frequent oral impairments reported by children already disadvantaged in terms of being Temeke residents and from a rural area must have been due to factors associated with material and social deprivation and could not entirely be attributed to various levels of oral diseases. Although not statistically significant in the multiple logistic regression analysis, the present results indicate a clear negative gradient with respect to dental visiting; the more frequent this habit, the less favorable the children's oral quality of life. This is consistent with results reported previously [34,35] and suggest that dental attendance may be recognized as a proxy for oral problems among Tanzanian school children. The results of the CFA confirm the three dimensions structure that the EFA identified namely the social-, physical-, and psychological-functional dimensions in the Kiswahili version of the Child-OIDP inventory. Those dimensions explain, respectively, 26%, 32% and 4% of the variance supporting the relative importance of the three domains of OHRQoL in this particular context. Factor analyses have been used previously with many other OHRQoL indicators to group their items into domains of various numbers, whereas some instruments consider OHRQoL as a single construct [32,36,37].

The Tanzanian Child-OIDP index exhibited marked floor effects in Kinondoni (81.5%) and Temeke (54.5%) but sufficient discriminative properties suggest that it is suitable for detecting group differences in cross-sectional studies. However, a substantial difference between the two districts occurred across the 8 aspects of daily living with 18.5% and 45.5% of children in Kinondoni and Temeke having experienced any oral impact during the 3 months preceding the survey. The observed prevalence is lower than those observed among similar age groups in other cultures [7,8,19], and also lower than those observed between older adolescents in Uganda as well as among Tanzanian adults of various ages [24,25,34]. Nevertheless, the prevalence rate of the Child-OIDP in Temeke compares with that obtained among UK children using the same instrument [20]. The higher prevalence of OIDP seen in Temeke as compared to Kinondoni is in line with Kinondoni children having a healthier profile generally both in terms of better clinical- and subjective oral health measures as well as socio-demographic characteristics such as parental education (Table 1). Eating was the most frequently reported impairment in both districts, a finding that is consistent with those of other populations using the adults and child versions of the OIDP instrument [6-8,19,20]. Although the prevalence of dental caries was low, toothache was recognized as the main cause of 6 out of 8 performances in Kinondoni and the main cause of 4 out of 8 performances evaluated in the Child-OIDP in Temeke where children reported a large range of oral problems as causes of oral impacts.

Further evaluation of the performance of children's OHRQoL instruments across countries and age groups has been requested. Validation of such instruments at the population level is important since clinical samples due to their biased nature may give a misleading picture. Further studies should assess its evaluative properties to determine its applicability to support clinical measures in oral health care intervention research.

## Competing interests

The author(s) declare that they have no competing interests.

## Authors' contributions

MM: Principle investigator, conceived of the study, designed the study, collected data, performed statistical analyses and manuscript writing.

ANÅ: Main supervisor, designed study, guided the statistical analyses. She has been actively involved in manuscript writing.

GT: He has provided valuable comments on the paper in general and on the OIDP scoring in particular. He has been actively involved in manuscript writing.

All authors read and approved the final manuscript.

## References

[B1] Slade GD, Assessing oral health outcomes (1997). Measuring oral health and quality of life : proceedings of a conference held June 13-14, at the University of North Carolina-Chapel Hill, North Carolina.

[B2] Slade GD, Strauss RP, Atchison KA, Kressin NR, Locker D, Reisine ST (1998). Conference summary: assessing oral health outcomes--measuring health status and quality of life. Community Dent Health.

[B3] Sheiham A, Spencer J, Pine CM (1997). Health need assessment. Community Oral Health.

[B4] Slade GD, Spencer AJ (1994). Development and evaluation of the Oral Health Impact Profile. Community Dent Health.

[B5] Adulyanon A, Sheiham A, Slade GD (1997). Oral impacts on daily performance. Measuring oral health and quality of life.

[B6] Astrom AN, Haugejorden O, Skaret E, Trovik TA, Klock KS (2005). Oral Impacts on Daily Performance in Norwegian adults: validity, reliability and prevalence estimates. Eur J Oral Sci.

[B7] Gherunpong S, Tsakos G, Sheiham A (2004). Developing and evaluating an oral health-related quality of life index for children; the CHILD-OIDP. Community Dent Health.

[B8] Gherunpong S, Tsakos G, Sheiham A (2004). The prevalence and severity of oral impacts on daily performances in Thai primary school children. Health Qual Life Outcomes.

[B9] Allen PF, Locker D (1997). Do item weights matter? An assessment using the oral health impact profile. Community Dent Health.

[B10] Kiwanuka SN, Åstrøm AN (2005). Self reported dental pain and associated factors in Ugandan schoolchildren. Norsk Epidemiology.

[B11] Jamil D, Åstrøm AN (2006). Prevelence and correlates of self-reported state of teeth among schoolchildren in Kerala, India. BMC Oral Health.

[B12] Gift HC, Reisine ST, Larach DC (1992). The social impact of dental problems and visits. Am J Public Health.

[B13] Kiwanuka SN (2006). Sugar snack consumption, caries experience and dental pain: surveys of 3-5 and 10-14-year-old children in Uganda. PhD Thesis..

[B14] Connolly MA, Johnson JA (1999). Measuring quality of life in paediatric patients. Pharmacoeconomics.

[B15] Jokovic A, Locker D, Stephens M, Kenny D, Tompson B, Guyatt G (2002). Validity and reliability of a questionnaire for measuring child oral-health-related quality of life. J Dent Res.

[B16] Jokovic A, Locker D, Stephens M, Kenny D, Tompson B, Guyatt G (2003). Measuring parental perceptions of child oral health-related quality of life. J Public Health Dent.

[B17] Foster Page LA, Thomson WM, Jokovic A, Locker D (2005). Validation of the Child Perception Questionnaire (CPQ 11-14).. J Dent Res.

[B18] Adulyanon S, Vourapukjaru J, Sheiham A (1996). Oral impacts affecting daily performance in a low dental disease Thai population. Community Dent Oral Epidemiol.

[B19] Tubert-Jeanin S, Pegon-Machat E, Gremeau-Richard C, M-M L, Tsakos G (2005). Validation of the French version of the Child-OIDP index. Eur J of Oral Sci.

[B20] Yusuf H, Gherunpong S, Sheiham A, Tsakos G (2006). Validation of an English version of the Child-OIDP index, an oral health-related quality of life measure for children. Health Qual Life Outcomes.

[B21] Badley EM (1987). The ICIDH: format, application in different settings, and distinction between disability and handicap. A critique of papers on the application of the International Classification of Impairments, Disabilities, and Handicaps. Int Disabil Stud.

[B22] Locker D (1988). Measuring oral health: a conceptual framework. Community Dent Health.

[B23] (2002). Ministry of Health. The United Republic of Tanzania:Oral Health Care Policy.

[B24] Kida IA, Astrom AN, Strand GV, Masalu JR, Tsakos G (2006). Psychometric properties and the prevalence, intensity and causes of oral impacts on daily performance (OIDP) in a population of older Tanzanians. Health Qual Life Outcomes.

[B25] Masalu JR, Astrom AN (2003). Applicability of an abbreviated version of the oral impacts on daily performances (OIDP) scale for use among Tanzanian students. Community Dent Oral Epidemiol.

[B26] (2004). National Bureau of Statistics.

[B27] Lwanga SK, Lemeshow S (1991). Sample size determination in health studies : a practical manual.

[B28] (1997). World Health Organization: Oral health surveys: Basic methods.

[B29] Greene JC, Vermillion JR (1964). The Simplified Oral Hygiene Index. J Am Dent Assoc.

[B30] Streiner DL, Norman GR, Streiner DL (1998). Selecting the items. Health measurement scales A practical guide to their development and use.

[B31] Byrne BM (2001). Structural equation modeling with AMOS : basic concepts, applications, and programming. Multivariate applications book series.

[B32] Bernabe E, Sheiham A, Tsakos G A comprehensive evaluation of the validity of the Child OIDP: further evidence from Peru. Community Dent Oral Epidemiol (submitted).

[B33] McDowell I, Newell C (1987). Measuring health : a guide to rating scales and questionnaires.

[B34] Astrom AN, Okullo I (2003). Validity and reliability of the Oral Impacts on Daily Performance (OIDP) frequency scale: a cross-sectional study of adolescents in Uganda. BMC Oral Health.

[B35] Masalu JR, Astrom AN (2002). Social and behavioral correlates of oral quality of life studied among university students in Tanzania. Acta Odontol Scand.

[B36] John MT, Hujoel P, Miglioretti DL, LeResche L, Koepsell TD, Micheelis W (2004). Dimensions of oral-health-related quality of life. J Dent Res.

[B37] John MT (2007). Exploring dimensions of oral health-related quality of life using experts' opinions. Qual Life Res.

